# Determination of Metal Impurities in Carbon Nanotubes Sampled Using Surface Wipes

**DOI:** 10.1155/2016/3834292

**Published:** 2016-11-16

**Authors:** Mary-Luyza Avramescu, Pat E. Rasmussen, Marc Chénier

**Affiliations:** ^1^Environmental Health Science and Research Bureau, HECSB, Health Canada, 50 Columbine Driveway, Tunney's Pasture 0803C, Ottawa, ON, Canada K1A 0K9; ^2^Department of Earth and Environmental Sciences, University of Ottawa, Ottawa, ON, Canada K1N 6N5

## Abstract

Residual metal impurities in carbon nanotubes (CNTs) provide a means to distinguish CNT from non-CNT sources of elemental carbon in environmental samples. A practical and cost-effective analytical approach is needed to support routine surface monitoring of CNT metal tracers using wipe sampling. Wipe sampling for CNT metal tracers is considered a qualitative indicator of the presence of CNTs, not a quantitative exposure metric. In this study, two digestion approaches (microwave-assisted nitric acid/H_2_O_2_ digestion and ultrasonic nitric/HF acid digestion) in conjunction with Inductively Coupled Plasma Mass Spectrometry (ICP-MS) determination were evaluated for their ability to extract metal impurities from CNT particles captured on wipe substrates. Aliquots of different carbon nanotubes (including NIST 2483 single-wall CNT) with and without GhostWipes™ (ASTM E-1792 compliant) were used to compare the performance of the digestion methods. The microwave digestion method accommodated the bulky wipe sample and also eliminated potential ICP-MS signal interferences related to incomplete digestion. Although quantitative recoveries requiring lengthy multistep digestion protocols may be necessary in other applications, the near-total recoveries achieved in the present study for CNT catalyst elements were adequate for identifying surface contamination of CNTs in the workplace using wipe sampling.

## 1. Introduction

Produced by various processes (i.e., cold vapor deposition, laser ablation, and arc discharge surface mediated growth), carbon nanotubes (CNTs) contain metal impurities (e.g., Fe, Co, Mo, Ni, and Y) [1–5] which are introduced in the manufacturing process as catalysts for carbon structure catalytic growth or as contaminants [2, 4–6]. It has been observed that even after rigorous purification those embedded impurities still persist in the CNT graphitic structure [2, 3, 6–9]. For example, an ICP-MS study of commercially important CNTs showed that impurity concentrations in three multiwall CNTs (MWCNTs) were between 4738 and 51867 *μ*g/g for Ni and in two commercially relevant single-wall CNTs (SWCNTs) between 1798 and 4217 *μ*g/g for Co and 1472−1672 *μ*g/g Mo [10].

The currently accepted method for determination of CNTs in workplace air is thermooptical analysis (NIOSH Method #5040) for determination of elemental carbon [11]. A drawback of this approach is that it does not distinguish between CNT and non-CNT sources of elemental carbon such as diesel exhaust [11, 12]. The detection of CNT-bound metal impurities in environmental samples provides a means to distinguish CNT emissions from non-CNT background sources of elemental carbon [12–15]. Worker exposure to CNTs can occur at any stage of the manufacturing process and during transport, storage, and handling of CNTs, with inhalation being the major route of exposure [11, 16]. Different sampling methods have been suggested as possible strategies to collect samples for workplace monitoring of metal impurities in CNTs such as filter cassettes, electrostatic precipitation, and surface wipes [13, 15]. Wipe sampling has emerged as a useful and cost-effective qualitative technique suitable for representative mapping of spatial variations of surface metal contamination within a workplace [17]. The method provides an additional tool to detect workplace releases of CNTs (and potential exposures), evaluate housekeeping or clean-up procedures, and monitor the effectiveness of engineering controls [15].

NIOSH [18] and OECD [17] provided guidance on collecting surface wipe samples in nanotechnology applications using premoistened GhostWipes towelettes, but neither included an analytical methodology for determining CNTs in wipe samples. Wipe sampling for CNT metal tracers is considered a qualitative indicator of the presence of CNTs, not a quantitative exposure metric [17]. As described by the OECD Tiered Approach [19], such qualitative assessments are useful for comparing particle concentrations at the emission source with background particle concentrations. Even though wipe sampling may be considered qualitative, an analytical method is needed that includes an efficient digestion step and adequate detection limits to ensure reliable and reproducible metal determinations. The most commonly used analytical methods for determining metal impurities in CNTs are Neutron Activation Analysis (NAA) [1, 3, 20–22], Direct Solid Sampling Electrothermal Atomic Absorption Spectrometry (DSS-ET AAS) [21], High Resolution Continuum Source Graphite Furnace Atomic Absorption Spectrometry (HR CS GFAAS) [9], Inductively Coupled Plasma Optical Emission Spectrometry (ICP-OES) [1, 4, 20, 21, 23, 24], and Inductively Coupled Plasma Mass Spectrometry (ICP-MS) [1, 3, 4, 7, 20, 21].

Out of all of these detection methods, ICP-MS is a suitably sensitive approach for determination of particle-bound metals on wipe substrates [25], but an appropriate extraction method is needed for CNT-bound metals. The key challenge is the difficulty in quantitatively extracting CNT metal impurities during the digestion step [3, 4, 7, 22, 26]. A variety of sample pretreatments have been employed for determination of metals in CNT samples including wet digestion [1, 3, 23], dry ashing combined with acid digestion [1, 3, 4], Carius tube digestion [22], microwave-assisted acid digestion [1, 3, 4, 20, 26], and microwave-induced combustion [4, 21, 24]. Grinberg and coworkers [4] reported incomplete recovery of metals from CNTs with the single-step microwave digestion (HNO_3_ and H_2_O_2_) recommended by Decker et al. [26] and proposed a multistep microwave-assisted procedure that involved the repeated use of strong acids as follows: HNO_3_ combined with H_2_O_2_ (1x), HNO_3_ alone (3x), evaporation near dryness, HNO_3_ and HClO_4_ followed by HNO_3_ addition and evaporation to dryness (4x), and finally dissolution and dilution in 1% HNO_3_. Grinberg et al. [4] found that this lengthy and aggressive procedure was necessary to ensure complete sample digestion and efficient recovery of residual catalysts (Co and Ni) and trace metal impurity content (Fe, Mo, Pb, and Hg) from the NRC SWCNT-1 reference material investigated. ISO/IEC 13278 [1] also recommended repeating the heating cycles with new reagent addition several times (i.e., three to six heating cycles depending on CNT structure) when using microwave digestion to measure CNT metal impurities. Yang et al. [20] emphasized the importance of the digestion step as they observed positive nonspectral interferences from undigested carbon residues remaining in the sample solution which they identified by comparing ICP-MS and ICP-OES results for the same SWCNT digest.

The purpose of this study is to identify an appropriate digestion approach for ICP-MS determination of metal impurities in CNTs collected using wipe samples. Two approaches are evaluated with respect to the efficiency of the extractions: a microwave-assisted digestion method and an ultrasonic digestion method. Although for this application 100% extraction efficiency is not required, the goal is to achieve reproducible and reliable recoveries that will enable the identification of CNT surface contamination. Different CNT materials, including NIST 2483 Soot (single-wall CNT), are digested with and without GhostWipes, as the digestion approach must be able to accommodate wipe samples which are quite bulky. The possibility that wipes may contain background contamination which can interfere with determination of the metals of interest is also considered.

## 2. Experimental

### 2.1. Materials

Three SWCNT products were obtained for this study: NIST 2483 SWCNT Standard Reference Material (Raw Soot; National Institute of Standards and Technology, Gaithersburg, MD, USA) and two other SWCNTs test materials, Aldrich-CNT (Sigma-Aldrich Co., Gillingham, UK) and a research test sample provided by National Research Council (Test-CNT). The NIST 2483-CNT is uniform and well-characterized carbon nanotube soot approx. 0.8 nm in diameter (by TEM) produced by a chemical vapor deposition variant process [27]. The CRM was weighed straight from the bottle, as the certificate of analysis indicates that the CRM does not require preparation prior to weighing. The Aldrich-CNT test material was manufactured by arc discharge, with 40–60 wt% carbon content and 30–35% metal content. This CNT has average diameter of 1.7 ± 0.2 nm, wall width of 0.4 ± 0.1 nm, and bundled dimensions of 5 to 10 nm in diameter and 0.32 to 4.7 *μ*m in length. The Test-CNT is a SWCNT with an average diameter and wall width of 1.6 ± 0.2 nm and 0.4 ± 0.2 nm, respectively, narrower bundles (diameter < 3 nm), and length between 0.07 and 5.2 *μ*m (high proportion < 1 *μ*m in length). The Test-CNT test material showed a higher proportion of amorphous material (relative to nanotubes) than that of Aldrich-CNT. Detailed characterization of Test-CNT and Aldrich-CNT test materials using SEM and TEM is provided in Rasmussen et al. [15]. NIST 1633b SRM (National Institute of Standards and Technology, Gaithersburg, MD, USA) is a bituminous coal fly ash sieved through a nominal sieve opening of 90 *μ*m (170 mesh) used for matrix comparisons and accuracy check. NIST 1633b SRM was used following the recommendations of Decker et al. [26].

### 2.2. Reagents and Solutions

High-purity nitric and hydrofluoric acids (SEASTAR Chemicals Inc., Sidney, BC, Canada) and Suprapur 30% aqueous solution of hydrogen peroxide were used for sample pretreatment. Ultrapure Milli-Q water (18.2 MΩ cm) was used for preparation of samples and calibration standards. High-purity multielement (MES-1107-01 Solutions A and B, 100 *μ*g/mL) and individual standard stock solutions (La and Gd, 1000 *μ*g/mL) were used to prepare the calibration standards (Delta Scientific Laboratory Products Ltd., Mississauga, ON, Canada). All standards were prepared in 1% HNO_3_ to match the matrix of the samples. GW procedural blanks and sample (NIST 2483) replicates were spiked at levels varying between 4 *μ*g/L and 250 *μ*g/L, respectively, with ICP-MSCS high-purity standard solution (10 *μ*g/mL). Individual high-purity standards solutions of Ge, In, and Re (1000 mg/L) were used to prepare the internal standard solution. Certified reference low and high level fortified waters for trace elements materials, TM-28.4 and TMDA 54.5, respectively, were used for quality control and were purchased from Environment Canada (Ottawa, ON).

GhostWipes (GW; Environmental Express, Charleston, South Carolina, SC 4250, premoistened with deionized water, individually sealed packets) were used in combination with NIST 2483 SWCNT and NIST 1633b STMs to assess the effect of GW on extraction efficiency. The present study used a 3.5 cm diameter circular disk of GhostWipes material as required by the wipe sampling device used for monitoring CNT releases. Composed of polyvinyl alcohol polymer, GhostWipes dissolve completely during the analytical procedure allowing the sample material/analytes to disperse in the digestion media (http://www.envexp.com/). They meet the ASTM E-1792 wipe sampling protocol. SCP Science DigiFILTERs 0.45 *μ*m (Teflon membrane) for 50 mL tubes were used for filtration of diluted extracts.

### 2.3. Instrumentation

Mettler Toledo XP205 digital analytical balance equipped with a U-shaped antistatic electrode was used to achieve accurate weighing results for the aliquots (2 mg) of tested CNTs used in this study. As recommended [1, 11], appropriate personal protective equipment was worn and care was taken to avoid inadvertent generation of aerosols when handling the CNT test materials.

An Ethos Touch Control Advanced Microwave Labstation (Milestone Microwave Laboratory Systems) equipped with Ethos TC built-in ATC-400-CE automatic temperature control was used for all microwave digestions. DigiPrep MS heating block (SCP Science, QC, Canada) and Branson ultrasonic bath (model B8510DTH) were used in this study.

For quantification of CNT metal impurities in digested samples, a NexION 300s Dual-Channel Universal Cell ICP-MS system (Perkin Elmer, Canada) coupled with a SC-Fast autosampler (Elemental Scientific, Omaha, NE) was operated in the standard and collision mode. The instrument was equipped with a high temperature apex-ST PFA MicroFlow nebulizer, cyclonic spray chamber with a PC3x chiller (2°C), and triple cone interface (nickel-platinum skimmer and sampler cone, and aluminum hypercone). The following conditions were used: plasma and auxiliary argon flow rates were 18 and 1.2 L/min, respectively. The nebulizer argon gas flow rate was 1.00 mL/min for the high temperature apex-ST PFA MicroFlow concentric nebulizer and the forward RF power was 1600 W. Optimization was carried out daily with a normal tuning solution. Three replicate readings were taken for all monitored masses and elements. The appropriate internal standards (Ge, In, and Re) and dilution factors were selected based on a preliminary semiquantitative analysis. Perkin Elmer Optima 5300V ICP-OES equipped with radial optical system (163 to 782 nm range) was used at the wavelengths recommended by manufacturer for the elements of interest. The instrument was operated at 1400 W power and flow rates of 15 L/min, 0.2 L/min, 0.8 L/min, and 1.00 mL/min for plasma, auxiliary, nebulizer, and peristaltic pump, respectively. Daily instrument tuning was done with a solution of 10 mg/L Mn (2% HNO_3_).

### 2.4. Digestion Methods

Two digestion methods were evaluated during this study to compare their efficiency in extracting elemental impurities from CNTs: a microwave digestion (MD) method using nitric acid and hydrogen peroxide and an ultrasonic digestion (UD) method using nitric and hydrofluoric acids. For both digestion methods three to five aliquots of 2 mg of test material were used for analysis (to mimic the particle mass on a surface wipe sample). Spiked samples (20 and 250 ppb level) and spiked procedural blanks (4 and 25 ppm level) were analyzed along with the test samples. Finally, NIST 2483 SWCNT and NIST 1633b Coal Fly Ash were combined with GhostWipes (GW) in order to compare the efficiency of investigated digestion methods in the presence of GW. Recoveries were calculated for each method and their advantages and disadvantages were considered.

#### 2.4.1. Microwave Digestion Method (MD, HNO_3_-H_2_O_2_)

The microwave digestion (MD) method was preceded by a DigiPrep sample preparation/pretreatment step. During the* DigiPrep step*, the samples were placed in 50 mL Teflon DigiTube vessels and were treated with diluted nitric acid (1 : 1, 12 mL) and allowed to sit at room temperature for 30–45 min. This step allowed the gentle and complete dissolution of GW; otherwise the generation of effervescence during heating may affect the sample and tube integrity. After effervescence subsided, the samples were treated successively at reflux (85°C on DigiPrep MS heating block) with diluted nitric (12 mL, 90 min), concentrated nitric acid (10 mL, 30 min reflux), and 30% hydrogen peroxide (5 mL, 45 min), and evaporated to 10 mL (90°C). The resultant acid-peroxide digestion solutions were then transferred quantitatively to precleaned high-pressure microwave digestion vessels (100 mL Teflon) and 0.5 mL 30% hydrogen peroxide. The* microwave digestion step *was performed at 1000 W power in the following conditions: 20 min to reach 180°C temperature, 10 min to increase from 180°C to 220°C, and 20 min at 220°C. The digested samples were evaporated to nearly dryness (90°C) and the resultant residues were dissolved in 1% nitric acid, vortexed, and filtered through a 0.45 *μ*m filter to remove the possible undissolved residuum (i.e., undissolved CNTs) that may impact the ICP-MS analysis. The DigiPrep pretreatment step was derived from McDonald et al. [25] (based on ASTM method E 1644) to accommodate the bulk of GW samples. Preliminary work [28] showed that the DigiPrep method alone did not yield adequate recoveries for CNTs in wipes and therefore it was necessary to add the microwave digestion step.

#### 2.4.2. Ultrasonic Digestion Method (UD, HNO_3_-HF)

The samples were placed in 15 mL digestion tubes with 4 mL nitric acid, 0.1 mL hydrofluoric acid, and 1.9 mL ultrapure water and allowed to stand at room temperature until GW dissolved completely and effervescence subsided (30–45 min) before proceeding to the next step. This step is most critical in the presence of GW because even with a small piece of GW (3.5 cm disc) an exothermic reaction develops during the hot water bath stage. The capped digestion tubes were placed in the ultrasonic water bath (180 W, 69°C) for a total of 2 h (2 × 1 h ultrasonication with 1 min vortex in between). The solutions were then cooled down, diluted to 10 mL using ultrapure water, and centrifuged (3500 rpm, 10 min). A final filtration step (0.45 *μ*m) was added to remove the possible undissolved residuum (i.e., undissolved CNTs) that may impact the ICP-MS analysis. All sample digests were analyzed by ICP-MS after appropriate dilution with 1% HNO_3_. The HNO_3_-HF UD method is based on a method originally designed for PTFE filter-based aerosol samples by Niu et al. [29].

All sample digests were stored in the fridge in precleaned polyethylene screw-capped tubes and analyzed by ICP-MS after appropriate dilution. The final digestion solutions were first screened for the presence of elements using ICP-MS and isotopes such as ^11^B, ^27^Al, ^47^Ti, ^51^V, ^52^Cr, ^55^Mn, ^57^Fe, ^59^Co, ^60^Ni, ^65^Cu, ^75^As, ^82^Se, ^88^Sr, ^66^Zn, ^89^Y, ^98^Mo, ^137,138^Ba, ^139^La, ^158^Gd, ^208^Pb, and ^238^U were monitored. Internal standard solutions of 25 ug/L Ge, In, and Re were used throughout the experiment for analytes in the mass ranges 87, 95–138, and 207–238, respectively. Analytical errors up to 10% for quality check standards were considered acceptable (EPA Method 200.8). Spike recoveries of the study elements (B, Al, V, Mn, Co, Ni, Cu, Mo, As, Ba, La, and U) were between 82% and 115% with MD method (except B 74% and Cu 47%; Al spike not recovered) and 86–107% with UD method (except Mo 122%) in the presence of GW (spiked procedural blanks and NIST 2483 sample). Aluminum, boron, zinc, and copper spike recoveries were affected by the presence of GW that contain variable amounts of contaminant elements introduced during their manufacturing and/or packaging. Therefore, five matrix blanks containing GW were included with each analytical batch and used for matrix blank corrections when samples were combined with GW. Limits of detection (LOD) are reported in Table 3. The LODs were calculated according to the 1994 US-EPA Guideline for Inductively Coupled Plasma Mass Spectrometry using 3 times standard deviation of 8-9 procedural blanks.

### 2.5. Statistical Tests

Data analysis was carried out with Sigma Plot 12.5 statistical software. Student's *t*-test and Mann-Whitney Rank Sum test were used for two sample comparisons.

## 3. Results and Discussion

Extraction efficiencies of two different methods, microwave digestion (MD) and ultrasonic digestion (UD), are compared for CNT impurities present at high concentrations (>0.5%) in Table 1 and for CNT impurities present in lower concentrations (ppm range) in Table 2 without GW being present. NIST 2483-CNT Standard Reference Material (SRM), Test-CNT, and Aldrich-CNT were used to compare the extraction efficiency for the residual catalysts (>0.5%). NIST 2483-CNT SRM was used to compare the extraction efficiency for other trace metal/element impurities (ppm range) using the certified, informational, or reference values provided in the certificate of analysis [27]. The catalyst residues in NIST 2483-CNT are Co and Mo with certified mass fraction values of 9630 ± 170 *μ*g/g and 34060 ± 290 *μ*g/g, respectively, determined by Instrumental Neutron Activation Analysis (INAA) and cold neutron Prompt Gamma Activation Analysis (PGAA) [27]. Certified values were given also for Ba, La, and Gd present in smaller quantities in NIST 2483-CNT, reference values for Al, Mn, and V, and informational values for the other metals (B, As, and Cu) as shown in Tables 2 and 3. Consequently, the efficiencies of the two investigated methods are compared using aliquots of NIST 2483-CNT combined with GW (Table 3).

### 3.1. Extraction of High and Low Concentration Metals from CNT Samples

Table 1 presents the high metal concentrations (>0.5%) in NIST 2483, Test-CNT, and Aldrich-CNTs obtained with both microwave and ultrasonic digestion methods. The results are presented as mean (*μ*g/g) and standard deviation of five independent determinations. As shown in Table 1 for all investigated CNTs, the MD method had improved extraction efficiency for the high concentration metals (Co, Ni, Y, and Mo) when compared to the UD method. Except for Ni in Test-CNT for which *t*-test indicated no significant difference (*p* = 0.128), for all other residual catalysts metals (Co, Ni, Y, and Mo) in all three CNTs the difference between MD and UD results was significant (*p* < 0.05). The greatest difference was observed for the NIST 2483-CNT (Table 1) for which 2–5 times lower Mo and Co concentrations were extracted with UD method than with MD method resulting in recoveries of 74 ± 2.0% Co and 82 ± 2.5% Mo with MD method and just 16 ± 1.7% Co and 39 ± 2.0% Mo with UD method. Although for Test-CNT (except for Ni) and Aldrich-CNTs the metal concentrations extracted with MD method were significantly higher (*p* < 0.05) than those obtained with UD method, the ranges were similar (i.e., Test-CNT Co: 17487 *μ*g/g MD versus 14358 *μ*g/g UD) when compared with results of NIST 2483-CNT (i.e., Co: 7100 *μ*g/g MD versus 1521 *μ*g/g UD). Overall, the results obtained with the UD method varied largely with the CNT analyzed, indicating that morphology and/or the way the residual catalyst metals were incorporated in the graphitic structure could influence the success of the sample pretreatment and consequently the recovery. This might be related to the manufacturing processes since the CNTs are produced by various processes, that is, cold vapor deposition, laser ablation, and arc discharge surface mediated growth [3, 4], which influence the amounts and how strongly the residual catalyst metals are incorporated in the graphitic structure [7, 22]. Even though in case of ultrasonic digestion method the recoveries vary with the CNT investigated, this proves to be a quick and useful method for qualitative identification of high concentration metallic impurities in CNTs.

Therefore, when comparing the two investigated methods, the MD method appears to be the method of choice for quantification of high concentration metals from tested CNTs due to improved extraction (Table 1) yielding high recovery of residual catalysts. The use of alternating oxidation and reflux steps with strong acids enhances the dissolution and extraction of metallic impurities from the CNT graphitic structure [30–34]. Overall, the MD extraction method takes 8 h-9 h (approx. 4.5 h for alternating oxidation and reflux steps plus evaporation and 4 h for microwave digestion and evaporation/filtration). In contrast, the UD method has a shorter sample-reagent contact time (2 h ultrasonication) and does not include alternating oxidation and reflux steps. Although the MD method takes more time than the UD method (and uses more reagents), the result is an increased extraction efficiency for high concentration metallic impurities in CNTs.

Beside the catalyst residues present in high concentrations (up to 3%) [2], lower concentrations of other metals are present in CNTs as a result of production procedures, postfabrication, and postpurification treatments [2]. Table 2 presents results obtained for lower concentration metal(loids)s in NIST 2483-CNT with both MD and UD methods. Generally both methods extracted similar amounts for most of the lower concentration (ppm range) metal(loids)s investigated. Even though slightly increased concentrations of Al, V, Mn, and Ba were extracted with the UD method and of Cu and La with the MD method, the differences were not significant (*p* > 0.05). In contrast, the MD method extracted significantly higher (*p* < 0.001) amounts of As, but significantly lower (*p* < 0.05) amounts of B, Gd, and U (Table 2).

**Figure 1:**
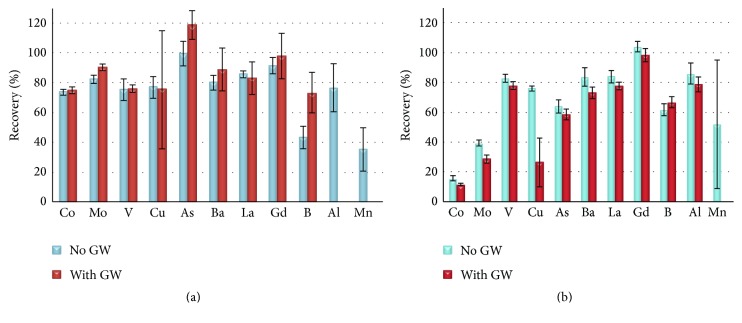
Percent recovery of certified value for NIST 2483 with (a) microwave digestion (MD) and (b) ultrasonic digestion (UD) methods with and without GhostWipes. The results are presented as mean (%) and standard deviation of five independent determinations.

Figure 1 shows the percent recoveries obtained for high (>0.5%) and low concentration (ppm range) metals in NIST 2483-CNT SRM obtained with (a) MD and (b) UD methods with or without GhostWipes (GW). Percent recoveries were calculated using the certified/information/reference values available in the certificate of analysis for the investigated elements [27]. Generally the recoveries for low concentration metals (i.e., Al, V, Cu, Ba, La, and Gd) ranged between 75 and 100% with MD and 76 and 104% with UD. Both methods resulted in low recoveries of B (43% MD and 62% UD) and Mn (35% MD and 51% UD), whereas UD gave also lower recoveries for As (64% UD versus 100% MD). With similar or increased recoveries (i.e., 85% Al, 83% V, and 84% La) for the low concentration (ppm range) metals investigated (except As) in NIST 2483-CNT, the UD method is a short and practical method that may be used to detect and measure metals present in lower amount in CNTs. Moreover, since NIST 2483-CNT is not certified for some elements of interest (i.e., Ni) and other metals present in higher concentrations were not well recovered with the ultrasonic digestion method (e.g., 16% Co), another SRM with carbonaceous matrix (as recommended by Decker et al. [26]), NIST 1633b, was analyzed using UD to assess the ability of the method to extract other metals, that is, Ni and Co when present in lower concentrations. This SRM was recommended previously for quality assurance in the absence of specific reference materials for CNT analysis [26]. It should be noted that elements in NIST 1633b are generally in lower concentration range (i.e., Ni: 120.6 ± 1.8 *μ*g/g; Co: 50 *μ*g/g) with the exception of Al and Fe (150.5 mg/g Al and 77.8 mg/g Fe). The certified values along with the results obtained for NIST 1633b SRM with UD method are presented in Table S1 (see Table S1 in the Supplementary Material available online at http://dx.doi.org/10.1155/2016/3834292). Recoveries of 78–91% were obtained with the UD method for most elements in NIST 1633b (with the exception of As, Zn, and Gd; Table S1) which were better than recoveries of 29–69% achieved for the same SRM using US-EPA method 3051 [35]. Even though the UD method showed excellent recoveries for low concentration metals in NIST 2483-CNT (i.e., Al, V, Cu, Ba, La, and Gd), recoveries were poor for the catalyst impurities (i.e., 16% Co and 39% Mo, resp.). Moreover, the extraction efficiency of the UD method for the residual catalysts/high concentration metals varied with the CNTs tested as shown in Table 1 with better extraction efficiency for Test-CNT and Aldrich-CNT than for NIST 2483-CNT. Compared to the MD method, the UD method results varied largely with the CNT analyzed, indicating that morphology and/or the way the residual catalyst metals were incorporated in the graphitic structure could influence the success of the sample pretreatment and consequently the recovery. This variability might be related to the manufacturing processes since the CNTs are produced by various processes, that is, cold vapor deposition, laser ablation, and arc discharge surface mediated growth [3, 4], which influence the amounts and how strongly the residual catalyst metals are incorporated in the graphitic structure [7, 22].

### 3.2. Effect of CNT-Derived Carbon Compounds on MD Extraction Results

**Figure 2:**
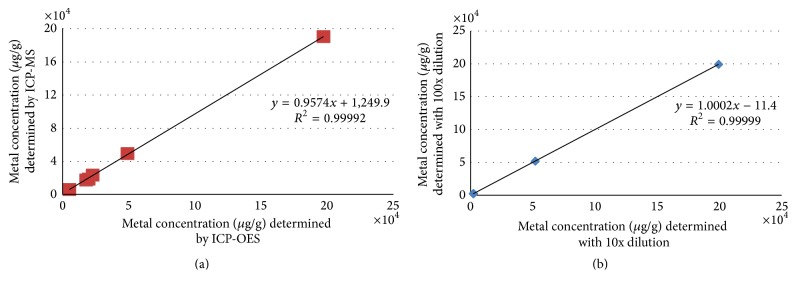
(a) Plot of metal concentrations in all three CNTs (NIST 2483, Test, and Aldrich) measured by ICP-MS and ICP-OES after microwave digestion (MD). (b) Plot of metal concentrations in spiked Aldrich-CNT 10 and 100 times diluted MD digests measured by ICP-MS. The straight line represents the best fit linear regression from (a) seven and (b) four pairs of concentration values (mean of five independent determinations).

Based on previous concerns about signal enhancement caused by nonspectral interferences [20, 36], the potential for signal enhancement was investigated for the MD method. This was done by analyzing the digests of all three tested CNTs (NIST-2483, NRC, and Aldrich) with both ICP-OES and ICP-MS techniques and comparing the results (Figure 2). The elements measured were Co, Ni, Y, Mo, and Fe (5 replicates of each). Concentrations of other elements were too low to be measured by ICP-OES. *t*-test results showed that the Co, Ni, Y, and Mo results obtained with ICP-MS were not significantly higher (*p* > 0.05) than those obtained with ICP-OES for all of the three CNTs tested, except for Ni in Aldrich-CNT (*p* = 0.031).  Figure 2(a) presents the correlation of metal concentrations measured by ICP-MS and ICP-OES using all seven pairs of metal-concentration values from all CNTs tested. The strong linear regression obtained (slope = 0.9574, *r* ^2^ = 0.99992, *n* = 7) indicates consistency between ICP-MS and ICP-OES results. Also, different dilutions (10x and 100x) of two spiked (200 ppb) Aldrich-CNT solutions obtained with MD method were analyzed (standard and/or collision mode) and the results compared for Co, Ni, Y, and Mo (Figure 2(b)). The results (slope = 1.0002, *r* ^2^ = 0.999998) presented in Figure 2(b) showed no differences between the ICP-MS results of the two dilutions. In summary, signal enhancement due to residual carbon does not occur with the MD/ICP-MS method and does not explain the higher recoveries observed with the MD versus the UD method.

### 3.3. Application of the Investigated Methods for Surface Wipes

Wipe sampling is commonly used in occupational safety and health applications [37]. Among other sampling methods (e.g., filter cassettes, electrostatic precipitation) surface wipes have been suggested as a strategy to collect samples (including CNT) for workplace monitoring [17, 37–39]. GhostWipes (GW) meet the US regulatory standard for sampling Pb in house dust and were used for quantification of additional metal(loid)s (i.e., As, Cd, Cr, Cu, and Ni) to provide information on typical background loadings for these metals in urban Canadian homes [25]. Although GW have the advantage of dissolving completely during acid digestion, their presence in the sample matrix adds to the analytical challenges encountered in the quantification of CNT metal bound impurities, since wipes are bulky and may contain impurities [25]. The MD method includes the DigiPrep sample pretreatment step that controls the dissolution of the bulky GW matrix (especially useful where the study design calls for one or two 15 × 15 cm GW per digestion tube).

The present study was designed for 3.5 cm discs of GW matrix, which permitted the use of smaller test tubes (15 mL) required by the ultrasonic method. The efficiencies of the two investigated methods were compared using aliquots of NIST 2483-CNT combined with GW (Table 3). NIST 1633b SRM was also analyzed with both methods with/without GW (Table S1, Supplementary Material). Results obtained in the presence of GW (Table 3) are significantly lower (*p* < 0.05) for Co and Mo in NIST 2483-CNT with the UD method than with the MD method, similar to results observed without GW (Table 1). Regarding lower concentration (ppm range) elements, the MD method extracted similar concentrations (*p* > 0.05) for most elements whether GW were present or not (except for B and As, *p* < 0.05). In contrast, with the UD method recoveries were significantly lower (*p* < 0.05) for almost all elements in the presence of GW than without GW (except B, Al, As, and Gd; *p* > 0.05) suggesting that the UD method is more sensitive to interferences from GW.

Problems are caused by high GW blanks for certain elements (Al, Ti, Ni, Mn, Zn, and Fe), which result in nondetects after blank correction, particularly when present in CNTs at lower concentrations. This is consistent with a previous study [25] which concluded that GW blanks were so elevated for Zn that residential Zn loadings could not be reported. Al, Ti, and Ni were recovered only with the UD method, and Mn, Fe, and Zn were not recovered with either of the two methods (Table 3). High blank values with poor recovery and/or nondetects after blank correction were also observed for certain elements (e.g., Al, Ti, Zn, Fe, and Mn) in our preliminary work with NIST 2483-CNT combined with whole GW [28] caused by uneven distribution of background contamination of these elements in the wipes used. (However, high blanks for certain elements would not necessarily be problematic if those particular metals are not target elements.) Other factors may include interferences from organic carbon derived from GWs or uncertainty arising from the small sample mass used in this study (2 mg versus 25–40 mg recommended in the certificate of analysis).

Figure 1 shows the same information as Tables 2 and 3, expressed as percentage recoveries (observed/certified value) for certified elements in NIST 2483. Combining the results into these histograms clarifies the overall trends: the MD method (a) yields acceptable recoveries for most elements with or without the presence of GW, but the UD method (b) yields poor recoveries for the catalyst elements and is generally more sensitive to the presence of GW. The UD method does result in acceptable recoveries (75–98%) for certain elements (V, La, and Gd) with or without the presence of GW. Despite generally lower recoveries, the cost-effective UD method may be considered acceptable for semiquantitative purposes such as identifying CNT releases in the workplace environment using wipe samples [15].

Different responses of the CNTs tested in the present study indicate that the CNT matrix may influence the success of the sample pretreatment and consequently the recovery. Thus, different matrices are likely to respond differently to a given digestion method, as observed for the UD method which gave excellent results for Co in NIST 1633b (Table S1, Supplementary Material) but poor recovery for Co in NIST 2483 (Table 1). Poor recoveries for the UD method (Figure 1(b)) are not likely due to readsorption of the extracted metals on residual CNTs, due to the strong acid digestion (pH < 2 throughout). Chen et al. [40] previously showed that the adsorption of metals on the surface of SWCNTs at pH less than 2 was negligible.

## 4. Conclusion

Wipe sampling for CNT metal tracers has emerged as a useful and cost-effective qualitative technique suitable for representative identification of CNT surface contamination in the workplace (and potential exposures) and routine monitoring of control measures [17]. Even though wipe sampling may be considered qualitative, an analytical method is needed that includes an efficient digestion step and adequate detection limits to ensure reliable and reproducible metal determinations. For the purpose of wipe sample analysis, the microwave digestion (MD) method yielded acceptable recoveries for catalyst elements at high concentrations (Table 1) as well as for elemental impurities at low concentrations (Tables 2 and 3) in the tested CNT reference materials. The MD method involved prolonged contact time between the reagents and acid, with alternating oxidation and acid reflux steps, which permitted the elemental impurities to be efficiently extracted from the CNTs. Moreover, no signal enhancement due to residual carbon compounds was observed when ICP-MS was used in conjunction with the MD method. Performance of the MD extraction was not affected by the presence of GhostWipes (GW) in the digest matrix, with the caveat that elevated and variable GW blanks for certain elements (such as Al and Zn) must be considered. An advantage of the MD method is that it eliminates the use of HF, which is avoided in many labs due to safety concerns. It is concluded that the MD method is appropriate for quantification of metallic tracers in wipe samples used to identify the presence of CNT in work environments.

In contrast, the ultrasonic digestion method showed incomplete extraction of catalyst metals present at high concentrations (Table 1), but near-total extraction efficiencies for elemental impurities present at low concentrations (Tables 2 and 3) in the tested CNTs. However, for NIST 1633b reference material, the UD method showed equivalent or better recoveries than those reported for US-EPA method 3051 [35], and since the UD method involves fewer steps than the MD method, it may serve as a cost-effective semiquantitative approach for screening purposes. For example, the UD method was useful for identifying the presence of CNT metal impurities (surface wipe samples) in a preliminary mapping survey of CNT releases in a manufacturing plant [15]. The UD method appeared to be more sensitive to interferences from GW than the MD method. Also, compared to the MD method, the extraction efficiency of the UD method varied to a greater degree with different carbonaceous matrices (various CNTs and coal fly ash). Nonetheless, CNT extraction efficiency is a parameter that can be evaluated using certified reference materials for CNTs, unlike other sources of laboratory and sampling uncertainty which are not as easily constrained (e.g., weighing errors, spatial and temporal variability).

## Supplementary Material

Table S1 Metal concentrations (μg/g) and percent recovery for NIST 1633b SRM obtained with MD and UD methods.

## Figures and Tables

**Table 1:** Metal concentrations (>0.5%) of NIST 2483, Test-CNT, and Aldrich-CNT obtained with both microwave digestion (MD) and ultrasonic digestion (UD) methods. The results are presented as mean (*μ*g/g) and standard deviation of five independent determinations. Certified values for Co and Mo in NIST 2483-CNT SRM and methods LODs are presented in Table 3.

Tested CNT	Element	Mean ± STDEV (*μ*g/g)	
		MD	UD
NIST 2483	Cobalt	7100 ± 196	1521 ± 163
	Molybdenum	28005 ± 852	13434 ± 693

Test-CNT^1,2^	Cobalt	17487 ± 1748	14358 ± 434
	Nickel	18457 ± 2005	15761 ± 1826
	Yttrium	23237 ± 1951	16746 ± 9703

Aldrich-CNT^1^	Nickel	184801 ± 4090	132745 ± 12664
	Yttrium	46283 ± 1205	25916 ± 4969

^1^MD (n = 4) and ^2^UD (n = 3).

**Table 2:** Metal concentrations (ppm range) of NIST 2483-CNT SRM obtained with both microwave digestion (MD) and ultrasonic digestion (UD) methods. The results are presented as mean (*μ*g/g) and standard deviation of five independent determinations.

Element	Certificate value	Mean ± STDEV (*μ*g/g)	
	(*μ*g/g)	MD	UD
Boron	74.7	32.2 ± 5.71	46 ± 2.88
Aluminum	723 ± 19	552 ± 116	621 ± 51.9
Vanadium	6.89 ± 0.14	5.19 ± 0.48	5.70 ± 0.18
Manganese	4.482 ± 0.041	1.57 ± 0.65	2.29 ± 1.91
Copper	186	143 ± 13.2	141 ± 2.94
Arsenic	12.5	12.5 ± 1.02	7.98 ± 0.57
Barium	119 ± 3.4	95.2 ± 5.92	99.5 ± 7.55
Lanthanum	104 ± 4	89.1 ± 2.47	87.4 ± 4.36
Gadolinium	10.6 ± 1	9.65 ± 0.58	11.0 ± 0.38
Uranium	Not certified	1.10 ± 0.07	1.80 ± 0.10

**Table 3:** Metal concentrations of NIST 2483-CNT with microwave digestion (MD) and ultrasonic digestion (UD) methods in the presence of GhostWipes. The results are presented as mean (*μ*g/g) and standard deviation of five independent determinations (nd = not detected due to blank correction; <LOD = below detection limit; n/a = not available).

Element	LOD (*μ*g/g)^*∗*^		Mean ± STDEV (*μ*g/g)		
	MD	UD	Certified value	MD	UD
Boron	9.72	12.4	74.7	54.7 ± 10.0	49.7 ± 2.89
Aluminum	431	26.6	723 ± 19	nd	568 ± 37.3
Vanadium	0.17	0.18	6.89 ± 0.14	5.25 ± 0.17	5.35 ± 0.17
Chromium	4.81	6.69	n/a	12.4 ± 7.07	12.3 ± 5.34
Manganese	3.87	0.25	4.482 ± 0.041	nd	nd
Iron	383	247	n/a	nd	<LOD
Cobalt	2.43	0.13	9630 ± 170	7206 ± 214	1059 ± 50.0
Nickel	2.65	0.97	n/a	nd	2.25 ± 0.85
Copper	7.54	2.52	186	140 ± 73.4	48.9 ± 30.5
Zinc	218	26.2	n/a	nd	nd
Arsenic	0.15	0.54	12.5	14.8 ± 1.21	7.32 ± 0.43
Molybdenum	2.71	5.96	34060 ± 290	30702 ± 795	9778 ± 905
Barium	1.19	0.32	119 ± 3.4	106 ± 16.8	87.0 ± 4.74
Lanthanum	0.15	0.07	104 ± 4	86.3 ± 11.3	80.8 ± 2.38
Gadolinium	0.04	0.06	10.6 ± 1	10.3 ± 1.60	10.4 ± 0.47
Uranium	0.02	0.06	n/a	0.93 ± 0.08	1.53 ± 0.11

^*∗*^Yttrium LOD: 5.12 *μ*g/g (MD) and 3.0 *μ*g/g (UD).
