# Supplementary material for: Determination of Metal Impurities in Carbon Nanotubes Sampled Using Surface Wipes
Source: J Anal Methods Chem. 2016 Nov 16;2016:3834292. doi: 10.1155/2016/3834292 (PMC5128706; doi:10.1155/2016/3834292)
Supplement: Supplementary file 1 — Table S1 Metal concentrations (μg/g) and percent recovery for NIST 1633b SRM obtained with MD and UD methods. [file 3834292.f1.pdf]

Determination of Metal Impurities in Carbon Nanotubes Sampled Using Surface Wipes

Mary-Luyza Avramescu, Pat E Rasmussen*, Marc Chénier. (*address correspondence to pat.rasmussen@hc-sc.gc.ca)

Supplementary Material

TABLE S1. Metal concentrations (μg/g) and percent recovery of NIST 1633b SRM obtained with microwave digestion (MD) and ultrasonic digestion (UD) methods. The results are presented as mean and standard deviation of three independent determinations. (nd=not detected due to blank correction; NIST 1633b SRM was not measured with MD method without GW).

Element	Certified value*	Mean ± stdev (μg/g)			Recovery (%,)		
		MD, GW	UD, GW	UD, no GW	MD, GW	UD, GW	UD, no GW
Boron		not measured	not measured	not measured	not certified	not certified	not certified
Aluminum	150500 ± 2700	57190 ± 17101	119143 ± 5159	124332 ± 4459	38 ± 11.4	79 ± 3.43	83 ± 2.96
Vanadium	295.7 ± 3.6	250 ± 39.5	244 ± 12.3	251 ± 7.57	85 ± 13.4	82 ± 4.16	85 ± 2.56
Manganese	131.8 ± 1.7	83.5 ± 9.81	109 ± 5.55	115 ± 2.38	63 ± 7.4	82 ± 4.21	87 ± 1.80
Iron	77800 ± 2300	66032 ± 12470	58747 ± 2476	61524 ± 989	85 ± 16.0	76 ± 3.18	79 ± 1.27
Cobalt	50	35.2 ± 4.30	41.0 ± 2.12	42.1 ± 1.18	70 ± 8.59	82 ± 4.24	84 ± 2.37
Nickel	120.6 ± 1.8	60.0 ± 24.5	98.9 ± 4.37	101 ± 3.18	50 ± 20.3	82 ± 3.62	83 ± 2.64
Copper	112.8 ± 2.6	73.6 ± 12.1	38.0 ± 20.5	96.5 ± 2.30	65 ± 10.7	34 ± 18.1	86 ± 2.04
Zinc	210	nd	nd	138 ± 4.98	nd	nd	66 ± 2.37
Arsenic	136.2 ± 2.6	130 ± 4.76	88.9 ± 5.06	89.7 ± 2.65	96 ± 3.49	65 ± 3.71	66 ± 1.94
Molybdenum		17.7 ± 1.90	15.9 ± 0.68	18.1 ± 2.61	not certified	not certified	not certified
Barium	709 ± 27	467 ± 62.1	591 ± 33.8	607 ± 19.0	66 ± 8.76	83 ± 4.77	86 ± 2.69
Lanthanum	94.0	52.5 ± 8.65	70.2 ± 3.86	73.0 ± 3.19	56 ± 9.20	75 ± 4.10	78 ± 3.39
Gadolinium	13.0	13.6 ± 1.84	18.0 ± 1.13	18.2 ± 0.54	104 ± 14.1	138 ± 8.71	140 ± 4.18
Lead	68.2 ± 1.1	54.5 ± 6.18	57.6 ± 2.80	58.9 ± 2.76	80 ± 9.07	84 ± 4.11	86 ± 4.05
Uranium	8.79 ± 0.36	6.01 ± 0.90	8.05 ± 0.34	7.98 ± 0.29	68 ± 10.2	82 ± 3.82	91 ± 3.24

* Co, Zn, La, Gd observational values
